# Lateral patellar retinaculum hypertrophy on magnetic resonance imaging predicts patellar subluxation in femoral trochlear dysplasia

**DOI:** 10.1002/jeo2.70475

**Published:** 2025-10-30

**Authors:** Zhaojun Huang, Weiyin Vivian Liu, Yu Pei, Xiaoping Huang, Yuanyuan Liu, Xingzhou Huang, Peng Zheng, Dong Liu

**Affiliations:** ^1^ Department of Radiology Songzi People's Hospital Jingzhou Hubei China; ^2^ MR Research GE Healthcare Beijing China; ^3^ Department of Radiology Hong'an County People's Hospital Huanggang Hubei China; ^4^ Department of Emergency, Tongji Hospital, Tongji Medical College Huazhong University of Science and Technology Wuhan Hubei China; ^5^ Department of Radiology, Tongji Hospital, Tongji Medical College Huazhong University of Science and Technology Wuhan Hubei China

**Keywords:** diagnostics, femoral trochlear dysplasia, lateral patellar retinaculum, patellar subluxation, the medial patellofemoral ligament

## Abstract

**Purpose:**

To explore the magnetic resonance imaging (MRI) features of the medial patellofemoral ligament (MPFL) and lateral patellar retinaculum (LPR) in femoral trochlear dysplasia (FTD) and assess their utility in predicting patellar subluxation (PS).

**Methods:**

Eighty patients with patellofemoral pain (30 controls, 32 FTD, 18 FTD + PS) underwent 3.0 T MRI. Axial T2‐weighted images were used to measure MPFL/LPR thickness and cross‐sectional area (CSA) at 5, 10 and 15 mm from the patellar attachment.

**Results:**

Significant differences in MPFL and LPR thickness and CAS existed among groups (all *p* values < 0.05). LPR metrics showed stronger correlation with lateral trochlear inclination (LTI) than MPFL at all points. LPR thickness at 15 mm correlated most strongly with LTI (*r* = −0.65). LPR CSA at 15 mm predicted PS (AUC = 0.80).

**Conclusions:**

LPR thickness and CSA—particularly from patellar attachment—represent potential predictive indicators for PS in FTD.

**Level of Evidence:**

Level III.

**Abbreviations**
AUC: area under curve
CNR: contrast noise ratio
CSA: cross‐sectional area
CT: computed tomography
FTD: femoral trochlear dysplasia
LPR: lateral patellar retinaculum
LTI: lateral trochlear inclination
MPFL: medial patellofemoral ligament
MRI: magnetic resonance imaging
PS: patellar subluxation
ROC: receiver operating characteristic
SD: standard deviation
SI: signal intensity
SNR: signal noise ratio
THK: thickness

## INTRODUCTION

Femoral trochlear dysplasia (FTD) is characterized by an abnormal morphology and insufficient depth of the trochlear groove, predisposing patients to patellar instability, cartilage wear and anterior cruciate ligament injuries [3, 4, 19, 21]. As the predominant aetiological factor for patellar instability, FTD exhibits >96% prevalence among symptomatic individuals [14, 26, 31, 32]. Severe instability may progress to patellar subluxation (PS), with a 33.6% recurrence rate following initial dislocation [11, 14, 22]. While primary patellar dislocation peaks at 10–17 years, chronic PS secondary to persistent FTD may manifest across all age groups [26]. Long‐term patellar instability accelerates cartilage degeneration and patellar softening, potentially leading to knee degeneration and even functional impairment [7, 9, 12, 15, 24]. The stability of the patellofemoral joint depends on the synergistic function of osseous structures and soft tissue structures, particularly the quadriceps muscle group, and the medial patellofemoral ligament (MPFL) and lateral patellar retinaculum (LPR) [8, 32]. The MPFL and LPR primarily stabilize the patella during early flexion (0–30°) by maintaining trochlear tracking, whereas bony constraints (trochlear geometry) dominate beyond flexion 30° [10, 35].

Computed tomography (CT) and magnetic resonance imaging (MRI) enable quantitative assessment of femoral trochlear morphology, including trochlear groove depth and angle, lateral trochlear inclination (LTI) and articular symmetry. LTI angle demonstrates exceptional clinical utility for evaluating groove depth and symmetry, achieving sensitivity and specificity of both reaching 100% and 96%, respectively [1, 18]. However, comprehensive FDT assessment requires supplementary evaluation of the lateral slope of the femoral trochlea beyond isolated morphological parameters.

In FTD patients, hypertrophy of the LPR correlates with the severity of LTI and predicts progression to PS. This study, therefore, aimed to explore the MRI features of the MPFL and LPR in FTD and assess their utility in predicting PS.

## MATERIALS AND METHODS

This study was approved by the institutional review board (IRB) of our hospital (IRB No. TJ‐RB202303157).

### Patients

A retrospective review was conducted on a total of 520 patients who underwent knee MRI examinations for patellofemoral pain at our hospital from January 2018 to August 2022. The inclusion criteria required the presence of patellofemoral pain and qualified MRI knee joint image data, and the exclusion criteria included patients with acute knee trauma (including patellar dislocation within 6 months, ligament ruptures or fractures) to prevent acute inflammatory effects on ligament morphology. Additionally, cases showing MRI evidence of acute soft‐tissue injury (e.g., T2 hyperintensity and fibre discontinuity) were eliminated. This dual exclusion criterion ensures observed morphological changes in stabilizers reflect chronic adaptive remodelling rather than acute healing responses.

### MRI examination

All subjects underwent an MRI examination on the 3.0 T Discovery MR750 scanner (GE Healthcare). The patient was positioned at the foot first with the knee joint at extension. Axial, sagittal and coronal fat‐suppressed T_2_‐weighted imaging (FS‐T_2_WI) sequences were performed with the following parameters: TR/TE = 3000/80 ms, slice thickness = 4.00 mm, spacing = 0.5 mm, echo‐train length = 14, matrix = 256 × 256 and FOV = 160 × 160 mm^2^. Additionally, sagittal T_1_‐weighted imaging (T_1_WI) was performed with the following parameters: TR/TE = 400/15 ms, slice thickness = 4.00 mm, spacing = 0.5 mm, echo‐train length = 3, matrix = 256 × 256 and FOV = 160 × 160 mm^2^.

### Case grouping

**Figure 1:**
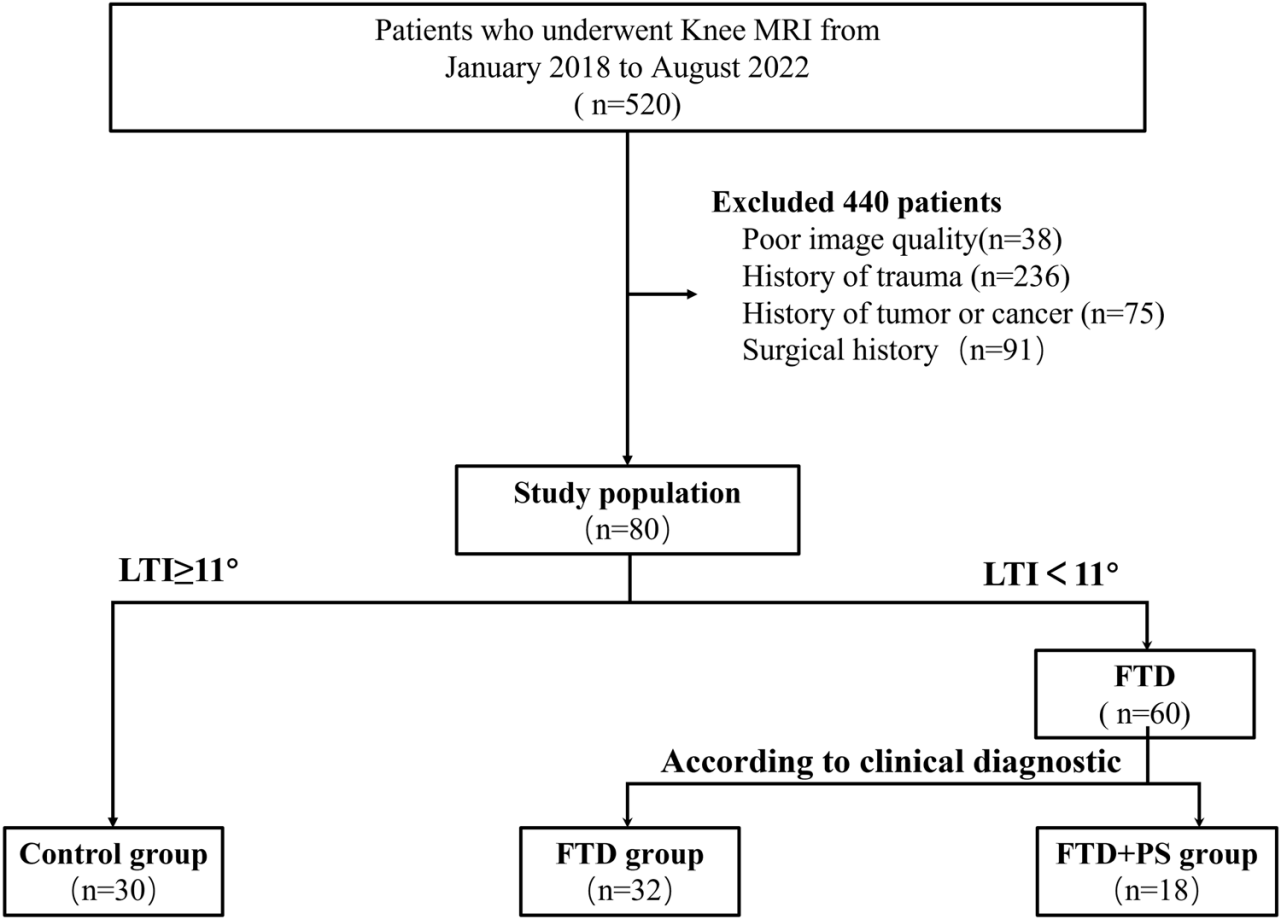
Flowchart of participant selection criteria. FTD, femoral trochlear dysplasia; LTI, lateral trochlear inclination; MRI, magnetic resonance imaging; PS, patellar subluxation.

The axial FS‐T_2_WI was used to diagnose FTD via quantitatively evaluating LTI and measuring the horizon distance at the proximal cartilage cross‐sectional area (CSA) of the trochlear groove [20, 29]. Eighty patients were divided into two groups: the FTD group (LTI < 11° as trochlear dysplasia) and the control group (LTI ≥ 11°), based on the trochlear inclination angle (angle < 11° as trochlear dysplasia). The FTD group was further divided into the FTD group and the FTD + PS group as lateral patellar shift (>50% patellar articular surface beyond the lateral femoral condyle edge) [17]. The flowchart of the participant selection criteria was presented in Figure 1.

### Objective evaluation of the medial and lateral ligaments

**Figure 2:**
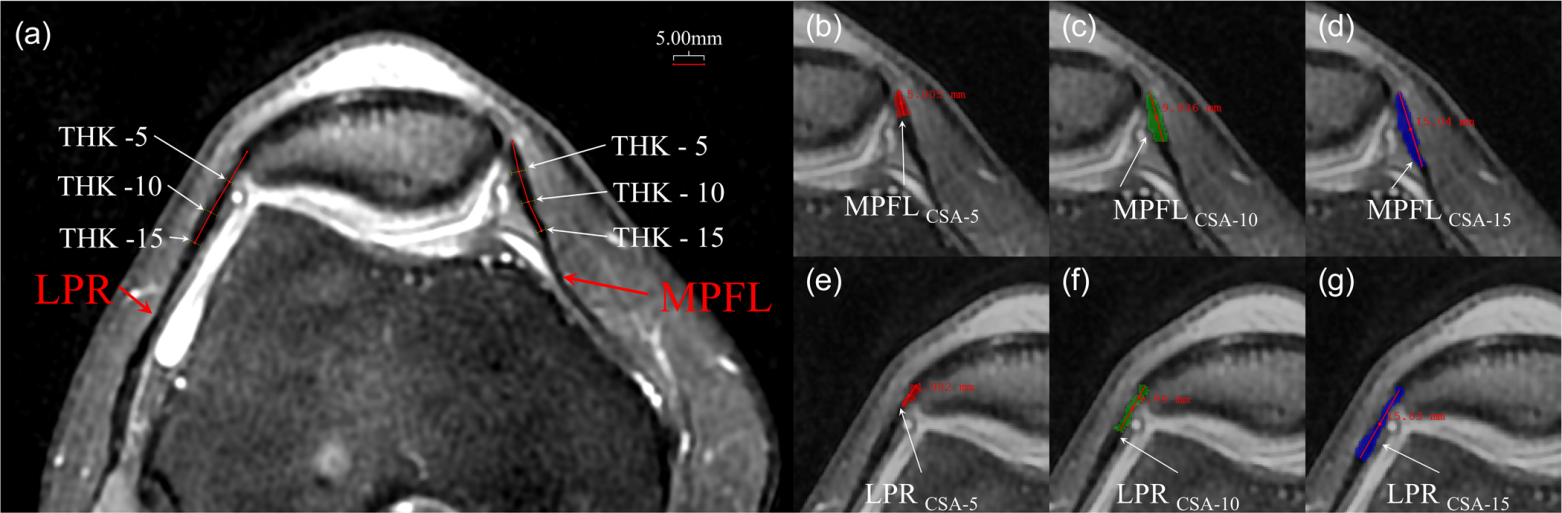
Illustrations demonstrate quantitative assessment of thickness (THK) (a) and cross‐sectional area (CSA) (b–g) for LPR and MPFL at standardized distances (5, 10 and 15 mm) from their patellar attachments, with CSA measurement regions denoted by dashed outlines. LPR, lateral patellar retinaculum; MPFL, medial patellofemoral ligament.

All measurements were performed on axial fat‐suppressed T2‐weighted images (FS‐T2WI) with the slice plane perpendicular to the patellar tendon, ensuring anatomical consistency. The patellar attachment was defined as the centroid of the insertion footprint on the medial/lateral patellar border. And the thickness (TNK), CSA and signal intensity (SI) of MPFL and LPR were measured at 5, 10 and 15 mm from patellar attachment along the ligament course [27] (MPFL_THK‐5_, MPFL_THK‐10_, MPFL_THK‐15_, LPR_THK‐5_, LPR_THK‐10_, LPR_THK‐15_, MPFL_CSA‐5_, MPFL_CSA‐10_, MPFL_CSA‐15_, LPR_CSA‐5_, LPR_CSA‐10_ and LPR_CSA‐15_, respectively). Distance calibration from patellar attachment points was performed using ITK‐Snap software (v3.8.0) with submillimeter precision. The formulas were as follows: signal noise ratio (SNR) = SI/SD_background_; contrast noise ratio (CNR) = (SI of ligament − SI of tibia)/(SD_background_), where SI and SD_background_ present signal intensity and standard deviation of background, respectively. Intra‐ and inter‐observer reliability of all MRI measurements (TNK, CSA and SI) was assessed using intraclass correlation coefficients (ICCs). Two musculoskeletal radiologists (with 5+ years of experience) independently measured 30 randomly selected knees. Measurements were repeated after a 4‐week interval to calculate intra‐observer ICC. Illustrations of measurements for the TNK and the CSA of LPR and MPFL at 5, 10 and 15 mm are shown in Figure 2.

### Statistical methods

Statistical analysis was performed using SPSS 22.0 (IBM). Data were expressed as the mean ± standard deviation (SD) and compared among the groups using one‐way analysis of variance (ANOVA), followed by the least significant difference or Dunnett's T3 test (determined by the homogeneity of variances test). Statistical significance was set *p* < 0.05. Pearson correlation coefficient was performed for continuous variables. Receiver operating characteristic (ROC) curve analysis was performed to evaluate the diagnostic efficacy of MPFL and LPR TNK, CSA and SI, and the optimal cut‐off values were obtained based on the Youden index (J). All area under the curves (AUCs) were compared using the DeLong test [36]. Inter‐observer ICC was derived from initial independent measurements. ICC values were interpreted as: poor (<0.50), moderate (0.50–0.75), good (0.75–0.90) and excellent (>0.90). The standard error of measurement (SEM) was calculated to quantify absolute measurement error using the formula: $\mathrm{SEM}=\mathrm{SD}\times \sqrt{1-\mathrm{ICC}}$.

## RESULTS

### Demographics

A total of 80 patellofemoral pain patients were divided into three groups: 30 cases in the control group (mean age ± SD: 40.83 ± 14.12), 32 cases in the FTD group (mean age ± SD: 38.72 ± 13.55) and 18 cases in the FTD + PS group (mean age ± SD: 35.33 ± 14.13) (*p* value = 0.418). There was no significant age difference among the three groups (*p* > 0.05). The FTD group and FTD + PS group had significantly lower inclination angle than the control group (*p* < 0.001), and there was no significant difference between the FTD group and FTD + PS group (FTD + PS vs. FTD, *p* = 0.96).

### Characteristics of MPFL and LPR

All quantitative MRI measurements demonstrated excellent intra‐ and inter‐observer reliability. ICC values ranged from 0.86 to 0.94 for intra‐observer reliability and 0.82–0.91 for inter‐observer reliability. The SEM was lowest for ligament TNK measurements (0.12–0.18 mm) and highest for CSA (12.3–18.7 mm^2^), consistent with the precision limitations of area quantification. SNR and CNR measurements showed moderate‐to‐excellent reliability (ICC: 0.78–0.89).

There were significantly different TNKs and CSAs of the MPFL and LPR (at 5, 10 and 15 mm) among the three groups (all *p* values < 0.05). Specifically, MPFL_THK‐5_, MPFL_THK‐10_ and MPFL_THK‐15_ showed no significant difference between the FTD + PS group and the FTD group (*p* values = 0.968, 0.948 and 0.305, respectively), and all were significantly thicker than those of the control group (all *p* values < 0.05). The TNK as well as CSA of the patellofemoral ligament at 10mm and 15 mm were the same, but the FTD group had a slightly lower CSA than the FTD + PS group at 5 mm (*p* value = 0.049) and no significant difference from the control group. Additionally, the TNK and CSA of the LPR at 5, 10 and 15 mm were significantly higher in the FTD + PS group than in the FTD and control groups (all *p* values < 0.05). Both CSA and TNK of LPR were from large to small in FTD + PS, FTD and the control groups, but there was no significant difference at the LPR 5 mm only between the FTD group and the control group (*p* value = 0.053). Furthermore, SNR and CNR of MPFL and LPR showed no significant difference among the three groups (SNR: *p* value = 0.112, *p* value = 0.052; CNR: *p* value = 0.440, *p* value = 0.283, respectively). All details are shown in Tables 1 and S1.

**Table 1:** Comparisons of clinical data and MRI features among the three groups.

Characteristics	Control (*n* = 30)	FTD (*n* = 32)	FTD + PS (*n* = 18)	*F*	*p*
Demographics					
Age (year)	40.83 ± 14.12	38.72 ± 13.55	35.33 ± 14.13	0.88	0.418
Gender^a^ (M:F)	14:16	16:16	10:8	N/A	0.837
BMI	22.86 ± 3.31	22.84 ± 2.71	21.77 ± 2.53	1.006	0.371
Morphometrics					
LTI (°)	18.63 ± 3.39	8.81 ± 1.98	8.85 ± 1.62	138.97	<0.001
MPFL_THK‐5_, mm	1.83 ± 0.54	2.60 ± 0.59	2.60 ± 0.62	16.59	<0.001
MPFL_THK‐10_, mm	1.85 ± 0.47	2.54 ± 0.56	2.55 ± 0.56	16.31	<0.001
MPFL_THK‐15_, mm	1.64 ± 0.44	2.28 ± 0.64	2.45 ± 0.61	14.68	<0.001
LPR_THK‐5_, mm	1.69 ± 0.38	2.55 ± 0.58	2.91 ± 0.72	32.71	<0.001
LPR_THK‐10_, mm	1.78 ± 0.44	2.65 ± 0.66	3.11 ± 0.69	31.84	<0.001
LPR_THK‐15_, mm	1.59 ± 0.38	2.48 ± 0.51	3.09 ± 0.69	52.80	<0.001
MPFL_CSA‐5_, mm^2^	67.06 ± 20.78	78.47 ± 21.04	91.81 ± 27.68	6.84	0.002
MPFL_CSA‐10_, mm^2^	127.94 ± 31.68	147.28 ± 33.10	161.97 ± 40.82	5.84	0.004
MPFL_CSA‐15_, mm^2^	196.66 ± 45.70	224.12 ± 43.39	250.55 ± 58.13	7.353	0.001
LPR_CSA‐5_, mm^2^	66.58 ± 18.56	78.36 ± 22.87	106.33 ± 31.23	16.17	<0.001
LPR_CSA‐10_, mm^2^	130.30 ± 37.19	153.81 ± 40.37	189.02 ± 36.79	13.16	<0.001
LPR_CSA‐15_, mm^2^	189.75 ± 52.20	227.56 ± 51.92	284.84 ± 50.29	19.07	<0.001
MRI characteristics					
MPFL signal	114.61 ± 51.24	176.57 ± 90.88	141.26 ± 67.90	5.61	0.005
LPR signal	109.37 ± 42.99	172.15 ± 81.26	150.77 ± 75.41	6.742	0.02
Tibia signal	64.58 ± 29.40	93.81 ± 55.20	82.24 ± 40.02	3.50	0.035
SD _background_	59.16 ± 21.00	80.82 ± 30.93	82.08 ± 35.30	5.56	0.006
SNR_MPFL_	1.93 ± 0.65	2.16 ± 0.73	1.77 ± 0.54	2.25	0.112
SNR_LPR_	1.84 ± 0.41	2.09 ± 0.35	1.86 ± 0.56	3.07	0.052
CNR_MPFL_	1.82 ± 0.60	2.03 ± 0.85	1.81 ± 0.63	0.829	0.440
CNR_MPFL_	1.75 ± 0.43	1.96 ± 0.51	1.89 ± 0.65	1.282	0.283

*Note*: Data presented as mean ± standard deviation (SD) unless specified.

Abbreviations: ANOVA, analysis of variance; BMI, body mass index; CNR, contrast noise ratio; CSA, cross‐sectional area; FTD, femoral trochlear dysplasia; LPR, lateral patellar retinaculum; LTI, lateral trochlear inclination; MPFL, medial patellofemoral ligament; MRI, magnetic resonance imaging; PD, patellar dislocation; PS, patellar subluxation; SNR, signal noise ratio; THK, thickness.

^a^ Chi‐square test for sex comparison, all others by one‐way ANOVA.

### Correlations between the MRI features of MPFL/LPR and the LTI

In Figure S1, MPFL_THK‐5_, MPFL_THK‐10_ and MPFL_THK‐15_ were moderately negatively correlated with LTI (*r* = −0.50, −0.47 and −0.49, respectively, all *p* values < 0.05). Additionally, MPFL_CSA‐5_ MPFL_CSA‐10_ and MPFL_CSA‐15_ were weakly negatively correlated (*r* = −0.31, −0.30 and −0.29, respectively, all *p* values < 0.05). LPR_THK‐5_, LPR_THK‐10_ and LPR_THK‐15_ were moderately to strongly negatively correlated with LTI (*r* = −0.54, −0.53 and −0.65, respectively, all *p* values < 0.05). LPR_CSA‐5_, LPR_CSA‐10_ and LPR_CSA‐15_ were weakly negatively correlated (*r* = −0.33, −0.36 and −0.37, respectively, all *p* values < 0.05).

Compared to MPFL, the TNKs and CSAs at the same positions of LPR had higher correlation with LTI, and were most correlated with the LPR_THK‐15_ and LPR_CSA‐15_ (Figures S1 and S2). In addition, there was no correlation between LTI, SNR and CNR of MPFL and LPR. The correlation coefficients between LIA, SNR and CNR of MPFL and LPR were weak (SNR: *r* = 0.00 and −0.15, *p* values = 1.00 and 0.18, respectively; CNR: *r* = 0.02 and −0.13, *p* values = 0.83 and 0.24, respectively).

### ROC analysis for the MRI features of MPFL and LPR

The AUC of the LTI in distinguishing the FTD + PS from PS groups was 0.52 (*p* value = 0.84). Compared to LPR, MPFL_THK_ and MPFL_S_ have AUCs of 0.51–0.66 in distinguishing between FTD + PS and PS (*p* > 0.05).

**Figure 3:**
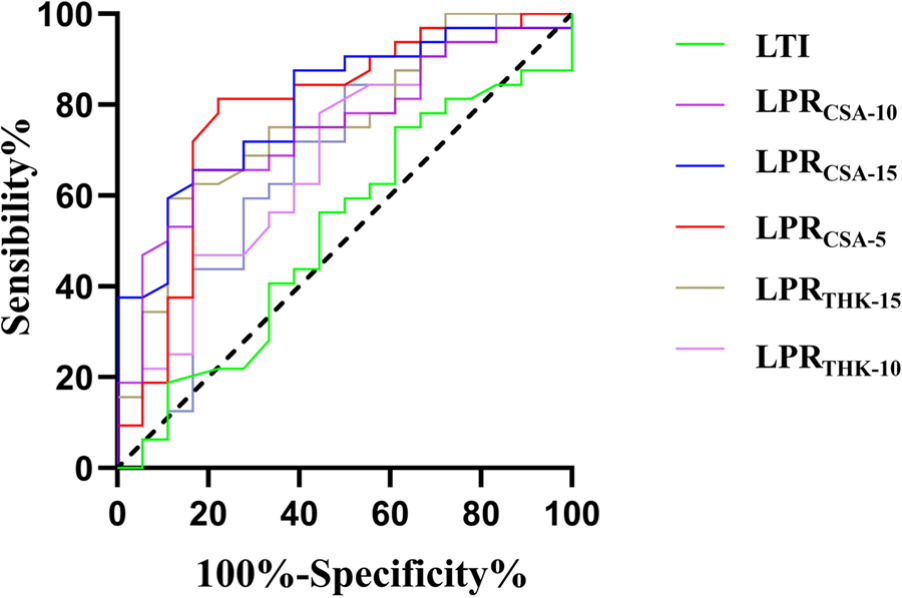
ROC curves evaluating lateral patellar retinaculum thickness (LPR_THK_), cross‐sectional area (LPR_CSA_) and LTI for identifying PS among FTD patients, highlighting LPR‐CSA at 15 mm as the optimal predictor compared to LTI. CSA, cross‐sectional area; FTD, femoral trochlear dysplasia; LPR, lateral patellar retinaculum; LTI, lateral trochlear inclination; PS, patellar subluxation; ROC, receiver operating characteristic; THK, thickness.

LPRs had good diagnostic value (AUC = 0.68–0.80, especially LPR_CSA‐15_ with the highest AUC of 0.8) in distinguishing FTD + PS from PS. The CSA at LPR_CSA‐5_ with the best sensitivity was 81.25%. The diagnostic value of LPR_THK_ was slightly inferior to LPR_S_, but LPR_THK‐15_ and LPR_CSA‐5_ both had the best specificity of 88.89% and positive predictive value (PPV) of 90.5% (Figure 3 and Table 2).

**Table 2:** Diagnostic efficacy of radiomics based on LPR and LTI on distinguishing between FTD + PS and FTD groups.

Parameter	LTI	LPR_THK‐10_	LPR_THK‐15_	LPR _CSA‐5_	LPR _CSA‐10_	LPR _CSA‐15_
AUC	0.52 (0.37–0.66)	0.68 (0.54–0.81)	0.76 (0.62–0.87)	0.78 (0.65–0.89)	0.75 (0.60–0.86)	**0.80** (0.66–0.90)
Cut‐off	8.46	3.15	2.5	86.64	154.8	228.8
Sensitivity (%)	75.00 (56.6–88.5)	78.12 (60.0–90.7)	59.38 (40.6–76.3)	81.25 (63.6–92.8)	65.62 (46.8–81.4)	65.62 (46.8–81.4)
Specificity (%)	38.89 (17.3–64.3)	55.56 (30.8–78.5)	88.89 (65.3–98.6)	77.78 (52.4–93.6)	83.33 (58.6–96.4)	83.33 (58.6–96.4)
PPV (%)	68.6 (58.9–76.8)	75.8 (64.4–84.4)	90.5 (71.4–97.3)	86.7 (72.9–94.0)	87.5 (70.7–95.3)	87.5 (70.7–95.3)
NPV (%)	46.7 (27.5–66.8)	5.8 (39.7–75.6)	55.2 (44.0–65.9)	70.0 (52.1–83.3)	57.7 (44.7–69.7)	57.7 (44.7–69.7)
Youden J	0.139	0.337	0.483	0.590	0.490	0.490

*Note*: Data in parentheses are 95% CIs. Bold word indicates the best values of the six predictors.

Abbreviations: AUC, area under the curve; CI, confidence interval; CSA, cross‐sectional area; FTD, femoral trochlear dysplasia; LPR, lateral patellar retinaculum; LTI, lateral trochlear inclination; NPV, negative predictive value; PPV, positive predictive value; PS, patellar subluxation; THK, thickness.

## DISCUSSION

This study indicated that THK and CSA of the MPFL and LPR_THK‐15_ moderately reflected the trochlear inclination angle. LPR_CSA‐5_ achieved a predictive accuracy of 0.80 for identifying FTD in PS patients, serving as a diagnostic foundation.

FTD causes patellofemoral pain and PS, with the progression potentially dominated by LPR and MPFL. LPR stabilizes the patellar stability by converging at the anterolateral fascial layer [13, 16, 28, 30], while MPFL stabilizes the lateral patellar displacement [5, 6]. During knee flexion, patellar articulation dynamically shortens and lengthens and thickens these ligaments [37], leading to soft‐tissue overload [33, 34]. Our finding of significantly increased THK of MPFL and LPR in patellar maltracking and PS groups compared to the control group suggested adaptive responses to mechanical demands. Histopathological studies have shown that altered mechanics induce secondary inflammation and fibrosis. FTD‐associated LPR hypertrophy reflects end‐stage fibrosis distinct from the acute healing process, as demonstrated by collagen disorganization and absent inflammatory infiltrates in histological studies [23, 25], whereas finite element analysis confirms that reduced lateral tilt of the trochlea increases the risk of patellar instability. Acute trauma cases (including recent dislocations with healing tissues) were excluded, and our cohort showed no ligament tears/oedema on MRI, ensuring findings reflect chronic adaptation, not healing.

Patellofemoral joint stability depends on soft‐tissue tensile strength and bony development. Early intervention may prevent degeneration. Five anatomical factors (trochlear depth/angle, Wiberg index, TT‐TG distance and Caton–Deschamps index) achieve 0.989 sensitivity for predicting recurrent PS [26]. Here, LPR CSA‐5 showed better predictive capability for PS in FTD patients than LTI (AUC = 0.78, sensitivity = 81.25%). This confirms MPFL/LPR thickening in FTD and positions LPR CSA‐5 as a potential PS predictor. The five anatomical risk factors, including trochlear depth, trochlear angle, Wiberg index, tibial tubercle‐trochlear groove distance and Caton–Deschamps index, achieve sensitivity of 0.989 for predicting recurrent PS [2]. In our study, the LPR_S‐5_ has better predictive capability for PS in FTD patients than LTI, with AUC and sensitivity of 0.78 and 81.25%. This suggested that the MPFL and LPR thickening in FTD and positions the LPR_S‐5_ as a potential PS predictor.

## LIMITATIONS

This retrospective study inherits limitations of non‐randomized designs, including selection bias (e.g., exclusion of trauma cases) and unmeasured confounders (e.g., activity levels). First, all measurements in static knee extension using standardized axial FS‐T2WI optimizes reproducibility, but failed to capture dynamic stabilizer function during flexion, where patellofemoral engagement occurs. Future protocols should incorporate 30° flexion positioning to simulate functional joint loading and assess trochlear containment efficacy. Second, our exclusion of acute traumatic patellar dislocations—which predominantly affect adolescents and young adults—resulted in a cohort skewed toward chronic FTD in middle‐aged patients, limiting applicability to paediatric or acute populations. While this design allowed characterization of adaptive soft‐tissue changes in established FTD, its limits direct applicability to acute paediatric/young adult dislocation populations. Chronic subluxation in FTD frequently persists into middle age, justifying inclusion of symptomatic older patients. Third, FTD subtypes (Dejour classification) were not analyzed due to sample size constraints (Dejour A/B/C/D require *n* ≥ 20 per subgroup). Future multi‐centre studies will address this. Finally, exclusion of significant distortion damage, ruptured patellofemoral ligament and traumatic dislocations in our study cohort precludes analysis of post‐traumatic instability to avoid acute confounding and thus necessitates comparing chronic traumatic versus atraumatic subluxation cohorts in future studies.

In conclusion, MPFL and LPR thicken in patients with FTD + PS and isolated FTD. LPR_CSA‐5_ predicts PS, and both MPFL and LPR ligaments critically maintain patellofemoral stability. These findings improve clinical risk assessment for PS.

## AUTHOR CONTRIBUTIONS

All authors contributed to the study conception and design. *Methodology*: Zhaojun Huang, Xiaoping Huang and Xingzhou Huang. *Formal analysis and investigation*: Weiyin Vivian Liu and Yuanyuan Liu. *Writing—original draft preparation*: Peng Zheng and Zhaojun Huang. *Writing—review and editing*: Dong Liu. *Funding acquisition*: Dong Liu. *Resources*: Yu Pei. *Supervision*: Dong Liu and Peng Zheng. All authors commented on previous versions of the manuscript. All authors read and approved the final manuscript.

## CONFLICT OF INTEREST STATEMENT

The authors declare no conflicts of interest.

## ETHICS STATEMENT

This study was approved by the Institutional Review Board (IRB) of Tongji Hospital (IRB No. TJ‐RB202303157).

## Supporting information

Supplementary file.

## Data Availability

The data that support the findings of this study are available in Supporting Information S1 of this article.
