# Supplementary material for: Lateral patellar retinaculum hypertrophy on magnetic resonance imaging predicts patellar subluxation in femoral trochlear dysplasia
Source: J Exp Orthop. 2025 Oct 30;12(4):e70475. doi: 10.1002/jeo2.70475 (PMC12573099; doi:10.1002/jeo2.70475)
Supplement: Supplementary file 1 — Supplementary file. [file JEO2-12-e70475-s001.docx]

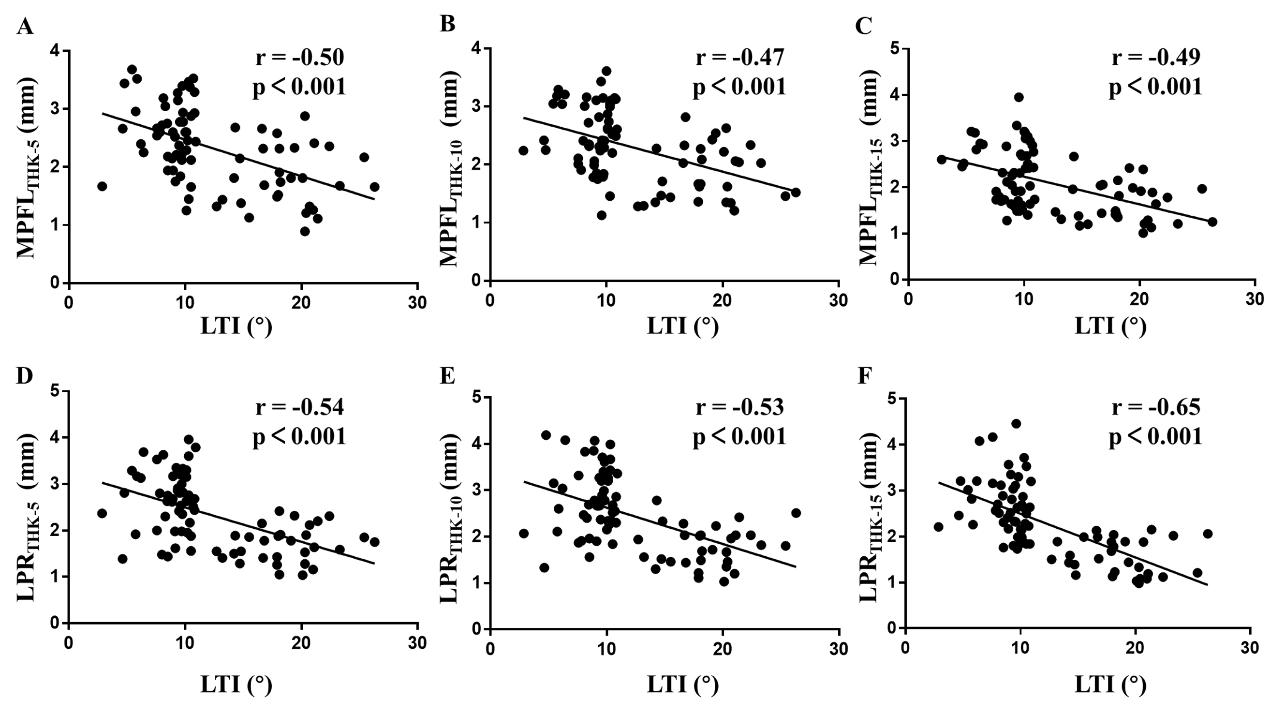


**Supplementary Figure 1.** Scatterplots for the MPFL_THK-5_ (A), MPFL_THK-10_ (B), MPFL_THK-15_ (C), LPR_THK-5_ (D), LPR_THK-10_ (E), LPR_THK-15_ (F) correlated with LTI, respectively.

LTI = lateral trochlear inclination; MPFL = medial patellofemoral ligament; LPR= lateral patellar retinaculum; THK= thickness.


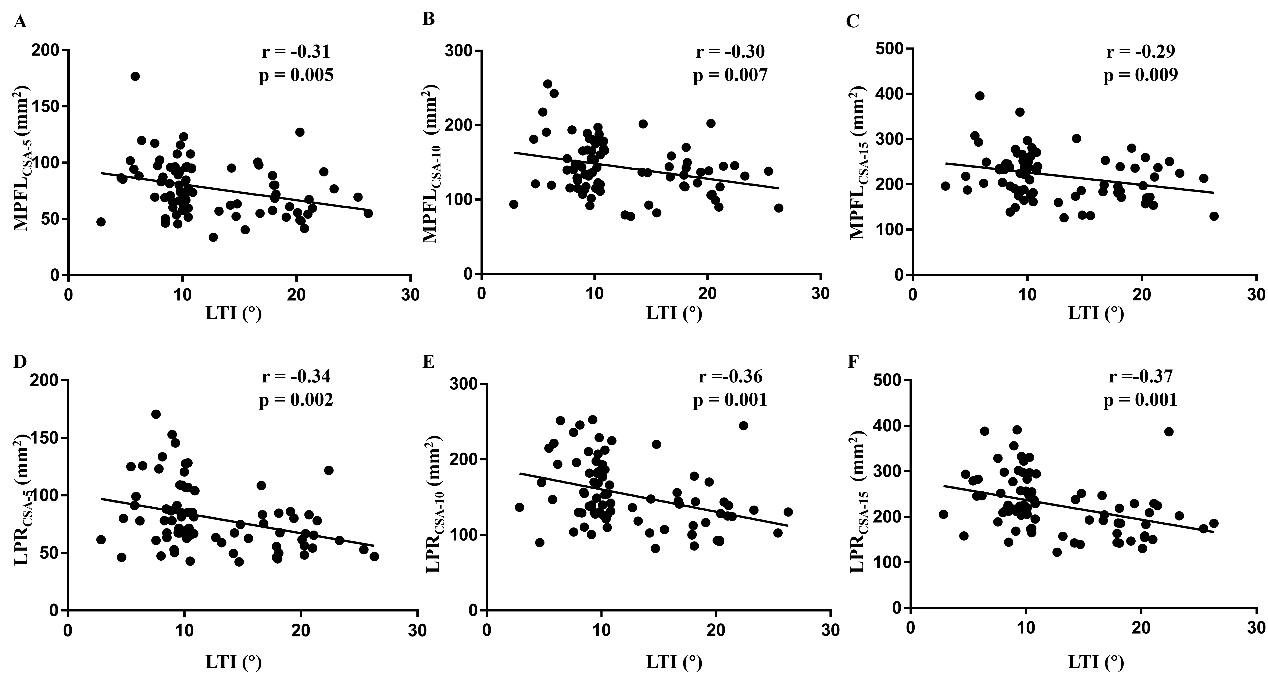


**Supplementary Figure 2.** Scatterplots for the MPFL_CSA-5_ (A), MPFL_CSA-10_ (B), MPFL_CSA-15_(C), LPR_CSA-5_ (D), LPR_CSA-10_ (E), LPR_CSA-15_ (F) correlated with LTI, respectively.

LTI = lateral trochlear inclination; MPFL = medial patellofemoral ligament; LPR= lateral patellar retinaculum; CSA = cross-sectional area.

Table S1. Statistical results of comparisons for clinical data and MRI features among three groups

| Characteristics | FTD+PS vs FTD | FTD+PS vs Control | FTD vs Control |
| --- | --- | --- | --- |
| **Demographics** |  |  |  |
| Age (year) | 0.411 | 0.188 | 0.551 |
| Gender† (M:F) | 0.774 | 0.766 | 0.805 |
| BMI | 0.196 | 0.971 | 0.213 |
| LTI (°) | 0.960 | ＜0.001 | ＜0.001 |
| **Morphometrics** |  |  |  |
| MPFL_THK-5_, mm | 0.968 | ＜0.001 | ＜0.001 |
| MPFL_THK-10_, mm | 0.948 | ＜0.001 | ＜0.001 |
| MPFL_THK-15_, mm | 0.305 | ＜0.001 | ＜0.001 |
| LPR_THK-5_, mm | 0.030 | ＜0.001 | ＜0.001 |
| LPR_THK-10_, mm | 0.010 | ＜0.001 | ＜0.001 |
| LPR_THK-15_, mm | ＜0.001 | ＜0.001 | ＜0.001 |
| MPFL _CSA-5_, mm^2^ | 0.049 | ＜0.001 | 0.050 |
| MPFL _CSA-10_, mm^2^ | 0.152 | 0.001 | 0.030 |
| MPFL _CSA-15_, mm^2^ | 0.065 | ＜0.001 | 0.027 |
| LPR _CSA--5_, mm^2^ | ＜0.001 | ＜0.001 | 0.053 |
| LPR _CSA--10_, mm^2^ | 0.003 | ＜0.001 | 0.018 |
| LPR _CSA-15_, mm^2^ | ＜0.001 | ＜0.001 | 0.005 |
| MPFL signal | 0.105 | 0.225 | 0.001 |
| **MRI Characteristics** |  |  |  |
| LPR signal | 0.289 | 0.044 | ＜0.001 |
| Tibia signal | 0.371 | 0.179 | 0.010 |
| SD | 0.882 | 0.009 | 0.004 |
| SNR_MPFL_ | 0.045 | 0.399 | 0.174 |
| SNR_LPR_ | 0.070 | 0.904 | 0.026 |
| CNR_MPFL_ | 0.311 | 0.972 | 0.258 |
| CNR_MPFL_ | 0.661 | 0.364 | 0.118 |

Note: LTI = lateral trochlear inclination; MPFL = medial patellofemoral ligament; LPR= lateral patellar retinaculum; FTD = Femoral Trochlear Dysplasia; PS = Patellar subluxation; THK= thickness, CSA = cross-sectional area; SD = standard deviation; SNR = signal noise ratio; CNR = contrast noise ratio
